# Adrenal Insufficiency Secondary to Bilateral Adrenal Hemorrhage: A Case Report

**DOI:** 10.7759/cureus.8596

**Published:** 2020-06-13

**Authors:** Waiel A Bashari, Yadee M. M Myint, Mya L Win, Samson O Oyibo

**Affiliations:** 1 Institute of Metabolic Science, Cambridge University Hospital, Cambridge, GBR; 2 Respiratory Medicine, Peterborough City Hospital, Peterborough, GBR; 3 Diabetes and Endocrinology, Peterborough City Hospital, Peterborough, GBR

**Keywords:** adrenal insufficiency, adrenal hemorrhage, adrenal apoplexy, case report, sepsis, spontaneous hemorrhage, cortisol, bilateral, corticosteroid replacement

## Abstract

Acute adrenal hemorrhage (adrenal apoplexy) in the context of severe sepsis is potentially life-threatening. Diagnosis of this condition is difficult to achieve without a strong sense of suspicion. The concurrent use of anticoagulants increases the risk of adrenal hemorrhage in the context of sepsis. Abdominal CT imaging is helpful in detecting hemorrhage within the adrenal gland. Once the diagnosis is considered, prompt therapy with corticosteroids can improve recovery and survival. A follow-up scan to confirm the resolution of the hematoma is useful to ensure that there is no other cause of adrenal enlargement. We report a 76-year-old lady who was hospitalized because of unexplained anemia and abdominal pain and was discovered to have bilateral pneumonia and urinary tract infection with severe hypotension not responding to standard treatments. An abdominal CT scan confirmed the presence of bilateral adrenal hemorrhage. A subsequent finding of an inappropriately low serum cortisol level in the presence of physiological stress confirmed adrenal insufficiency. The patient’s condition improved following corticosteroid replacement. A repeat CT scan performed 10 months following the patient’s initial presentation demonstrated signs of resolution of the adrenal hematomas; however, the patient’s adrenal function remained impaired.

## Introduction

Adrenal hemorrhage (adrenal apoplexy) is a term used to describe acute bleeding into the adrenal gland. It is similar and also referred to as Waterhouse Friderichsen Syndrome (WFS), which occurs in the setting of severe sepsis usually caused by meningococcal bacteremia [1]. It is a potentially fatal condition if not promptly diagnosed and treated. Case reports of acute adrenal hemorrhage are relatively scarce, possibly indicating a degree of under-diagnosis or misdiagnosis. It is essential to consider the diagnosis in states of severe sepsis associated with circulatory failure. Also, the presence of factors that increase the risk of bleeding should alert clinicians to consider this diagnosis [2]. Abdominal imaging using CT is a useful tool in detecting hemorrhage [3]. In severely ill patients suspected of having adrenal insufficiency, a stimulated increment in the cortisol level below 248.3 nmol/L (after administration of 250 µg of cosyntropin® or synacthen®) or a random total cortisol level below 275.9 nmol/L is diagnostic [4]. Treatment with corticosteroids should be commenced as soon as the diagnosis is considered: affected patients will require long-term follow-up [4]. We present a case of spontaneous bilateral adrenal hemorrhage in a 76-year-old lady who was initially hospitalized due to unexplained anemia and abdominal pain and found to have bilateral pneumonia, urinary tract infection, and intractable hypotension.

## Case presentation

Medical history and demographics

A 76-year-old lady was admitted to hospital when she was discovered to have severe anemia (hemoglobin level of 61 g/L, packed cell volume of 0.191 L/L, mean cell volume of 94.6 fl) while on oral anticoagulant therapy (prothrombin time ratio of 3.7). She had a four-week history of non-specific left-sided abdominal pain, nausea, reduced appetite, passing dark stools, and breathlessness on exertion. The patient was given 10 mg of oral vitamin K and two units of packed-cell blood transfusion followed by an esophagogastroduodenoscopy, which did not reveal any source of upper gastrointestinal bleeding or abnormality. Four days later while an inpatient, she became acutely confused and unwell with symptoms and signs of a chest infection including fever and severe hypotension, which did not respond to intravenous antibiotics and intravenous fluids. Further investigations revealed results, which prompted endocrinology assessment. The patient’s past medical history consisted of hypothyroidism for which she took levothyroxine 125 µg daily and systemic lupus erythematosus (SLE) for which she took warfarin tablets 3 mg daily for previous cranial vasculitis and thromboembolic episodes. She had been tried on hydroxychloroquine in the past but this was stopped because of visual side effects, and her SLE was under control. Apart from that, the patient was usually fit and well. There was no past history of prolonged high-dose corticosteroid usage, but she had received a short course (prednisolone 10 mg daily for one week) two years prior to this presentation.

On the day of admission, she was pale and had mild tenderness in the left iliac fossa on abdominal examination. The rectal examination did not reveal any source of bleeding. Repeat examination demonstrated a heart rate of 98/min and hypotension (blood pressure 75/50 mmHg). She had a temperature of 38°C, respiratory rate of 18 breaths per minute with an oxygen saturation of 95% on room air. Chest examination revealed dull notes and inspiratory crackles on both bases. Abdominal examination revealed no abnormal findings.

Investigations

Routine blood tests demonstrated features in keeping with infection (raised C-reactive protein, thrombocytopenia, and raised D-dimer). The coagulation profile indicated mild anticoagulation due to the warfarin (the initial raised prothrombin time ratio corrected by vitamin k administration). An arterial blood gas sample demonstrated type 1 respiratory impairment (Table 1). Blood cultures did not yield any organism but a urine culture yielded clinically significant urinary tract infection by the *Enterococcus faecalis* bacteria sensitive to amoxicillin and nitrofurantoin.

**Table 1:** Results of routine hematological and biochemical investigations These are results of subsequent blood tests done when the patient became confused and hypotensive

Blood parameters	Normal range	Patient’s results
Hemoglobin (g/L)	115-165	109
White cell count (10^9^/L)	4.0-11.0	8.0
Eosinophil count (10^9^/L)	0-0.5	0
Platelets (10^9^/L)	150-400	91
Prothrombin time ratio (INR)	0.8-1.25	1.23
Activated partial thromboplastin time (APTT) ratio	0.8-1.2	1.87
D-dimer (ng/ml)	< 243	701
Sodium (mmol/L)	133-146	131
Potassium (mmol/L)	3.4-5.1	3.9
Creatinine (µmol/L)	45-84	74
Adjusted calcium (mmol/L)	2.2-2.6	2.11
Urea (mmol/L)	2.5-7.8	3.8
C-reactive protein	< 10	196
9 am cortisol (nmol/L)	250-600	23
Alkaline phosphatase (U/L)	30-130	253
Bilirubin (µmol/L)	< 21	6
Albumin (g/L)	35-50	28
Alanine transferase (ALT)	10-60	26
Amylase (U/L)	0-100	25
Arterial blood pH	7.35-7.45	7.425
Arterial partial pressure of carbon dioxide (kPa)	4.67-6.0	4.2
Arterial partial pressure of oxygen (kPa)	10.67-13.33	9.66

**Figure 1:**
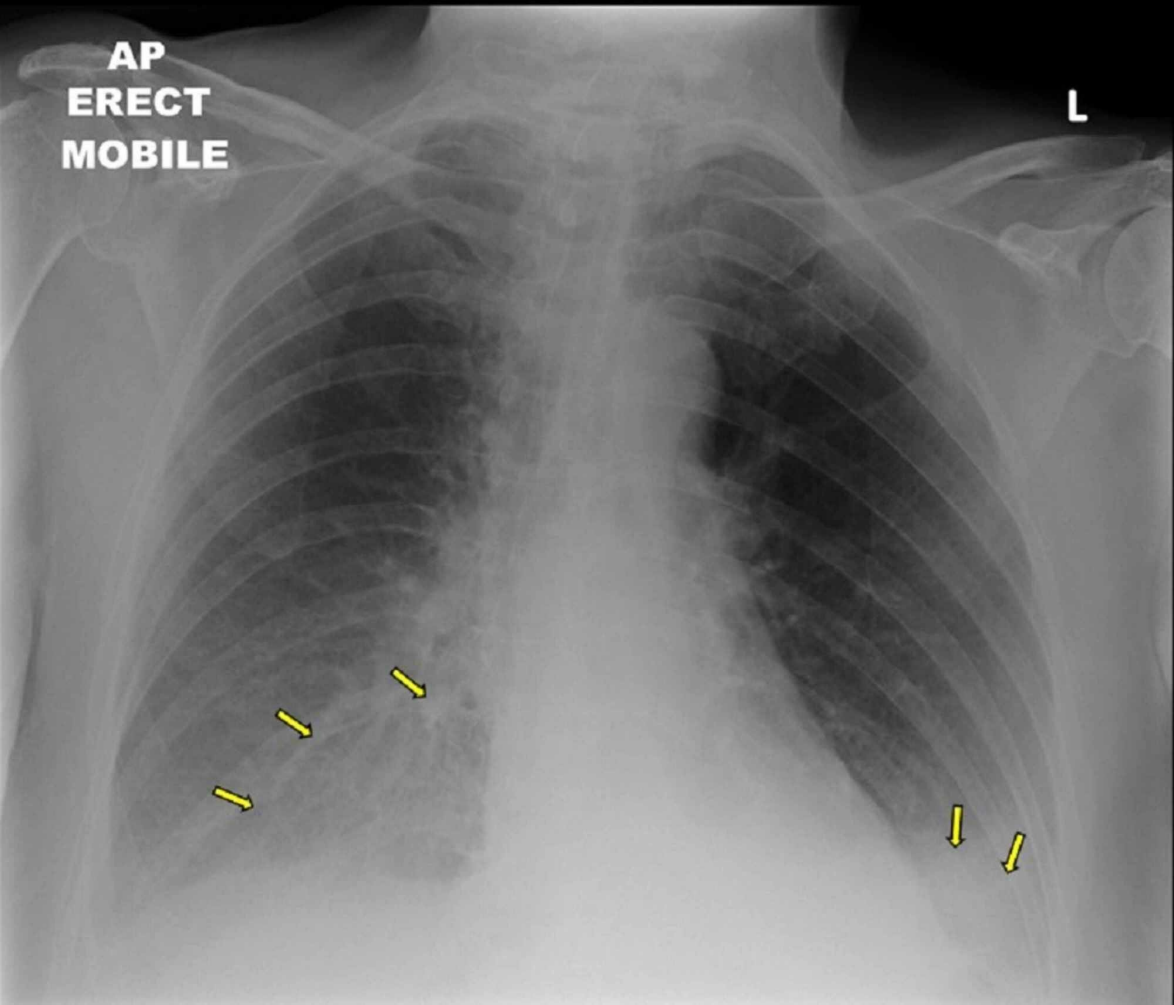
Chest x-ray showing bilateral basal pneumonia (arrows)

A standard chest x-ray showed evidence of bilateral basal pulmonary basal infiltrates in keeping with bilateral basal pneumonia (Figure 1). Sputum sample analysis did not detect any causative organism.

**Figure 2:**
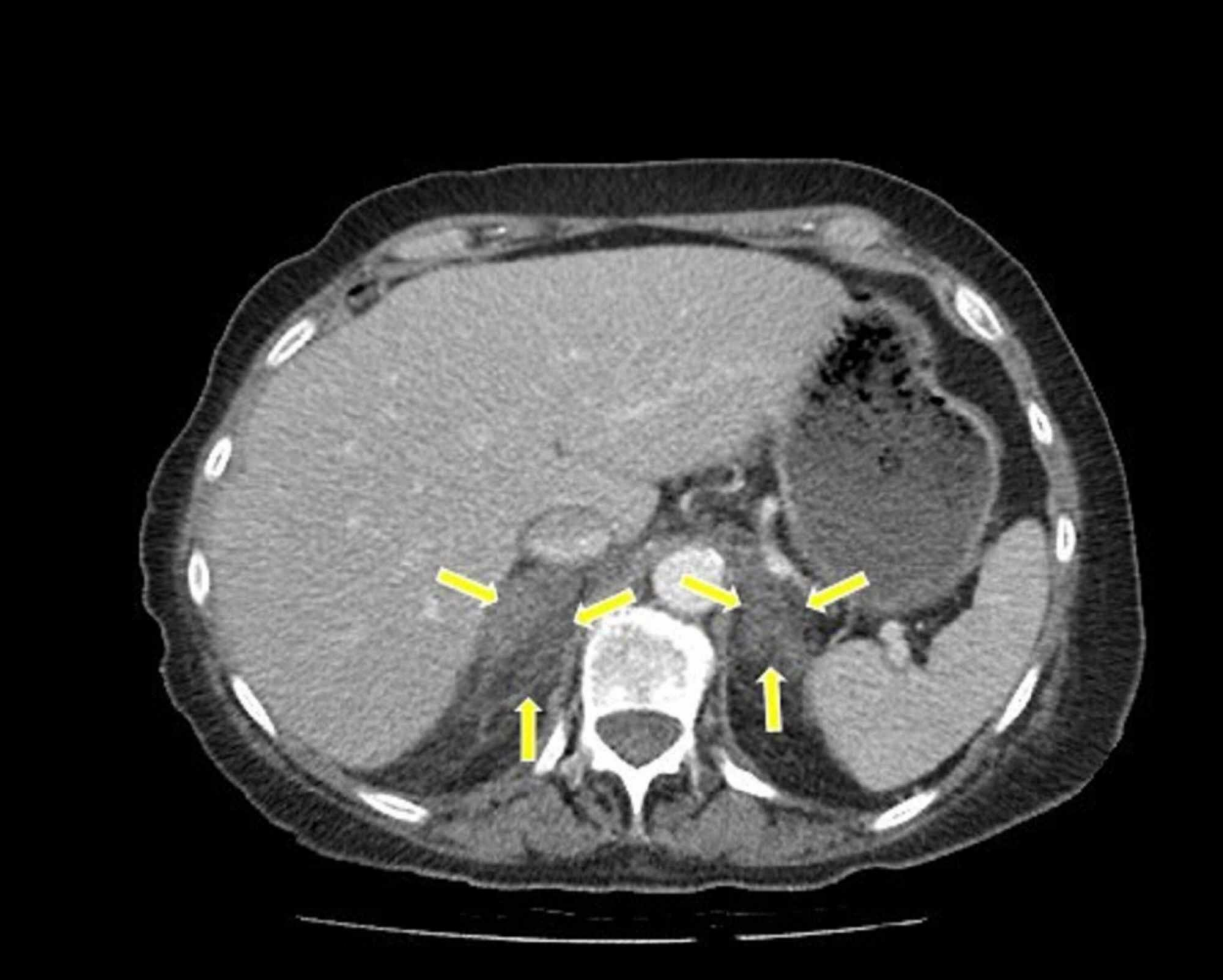
CT scan demonstrating bilateral adrenal hemorrhage (arrows)

Because of the unexplained abdominal pain, anemia and intractable hypotension, a CT scan of the chest, abdomen and pelvis was performed to look for any source of sepsis or blood loss. The abdominal CT scan revealed large bilateral adrenal shadows with a lack of adrenal gland configuration due to large adrenal hematomas (Figure 2). The chest CT scan confirmed the previous chest x-ray findings.

A random cortisol assay performed on a morning blood sample revealed severe hypocortisolemia (serum cortisol of 23 nmol/L) in the presence of severe physiological stress and sepsis. This result was a diagnostic of adrenal failure.

Treatment

The patient was continued on intravenous antibiotics and fluid resuscitation for sepsis related to bilateral pneumonia and urinary tract infection. However, the blood pressure rapidly improved towards the normal range only once 100 mg of intravenous hydrocortisone was administered as an immediate single dose followed by 50 mg four times a day [4]. Once the patient’s condition improved, the intravenous hydrocortisone regimen was changed to oral hydrocortisone 20 mg daily (10 mg in the morning, 5 mg midday, and 5 mg in the evening). Oral fludrocortisone 100 mcg daily was added for mineralocorticoid replacement. The anticoagulant medication (warfarin) was discontinued following this episode. The patient had a hospital stay of 14 days. Upon discharge, the patient was given instructions regarding steroid replacement therapy, the steroid ‘sick-day’ rules, a steroid identification card, and an intramuscular (IM) steroid kit for emergency use.

Outcome and follow-up

**Figure 3:**
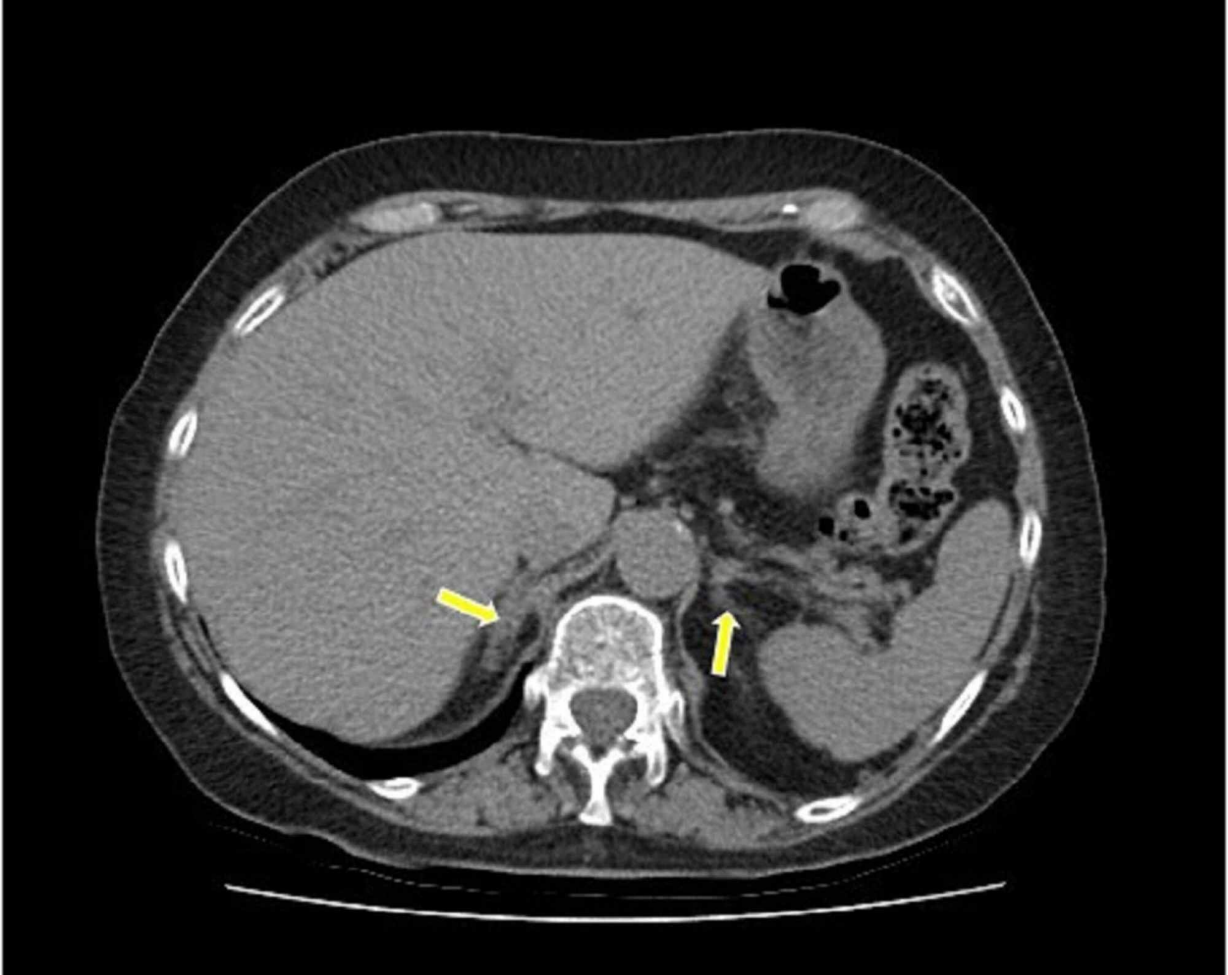
Follow-up CT scan demonstrating resolution of the bilateral adrenal hematomas

The clinical outcome was very satisfactory. Nine months following discharge, a repeat CT scan demonstrated a marked degree of resolution of the bilateral adrenal hematomas (Figure 3). The patient remained clinically stable on her corticosteroid replacement therapy and inquired about complete recovery. A follow-up short Synacthen® test done at 10 months and another at two years after presentation confirmed on-going adrenal insufficiency (Table 2). The patient remains on long-term corticosteroid replacement therapy, and has been started on a direct oral anticoagulant.

 

**Table 2:** Results of the two short Synacthen® tests

Time after presentation	Cortisol sample	Normal range (nmol/L)	Patient’s results
10 months	0-minute	250-600	31
	30-minute	> 550	30
2 years	0-minute	250-600	28
	30-minute	> 550	27

## Discussion

Sepsis-associated adrenal hemorrhage is considered an uncommon cause of adrenal insufficiency during acute critical illness. A post-mortem series have demonstrated a prevalence of around 1.1% in patients treated for sepsis [5]. Bleeding within the adrenal cortex causes compression and destruction of all three layers resulting in the deficiency of the adrenal hormones (glucocorticoids, mineralocorticoids, and adrenal androgens) [5].

Both the etiology and pathogenesis of adrenal hemorrhage are largely unknown; however, several mechanisms have been speculated. Bacterial infection has repeatedly been implicated as an important contributor in the etiology of spontaneous adrenal hemorrhage [6-8]. Coagulopathy leading to increased bleeding risk, in some cases, disseminated intravascular coagulation (DIC) is a recognized complication of severe sepsis. The deranged coagulation, together with the increased endothelial permeability associated with sepsis, may contribute to the etiology of adrenal hemorrhage [9]. The concurrent use of anticoagulants is another recognized risk factor for spontaneous adrenal hemorrhage.

**Figure 4:**
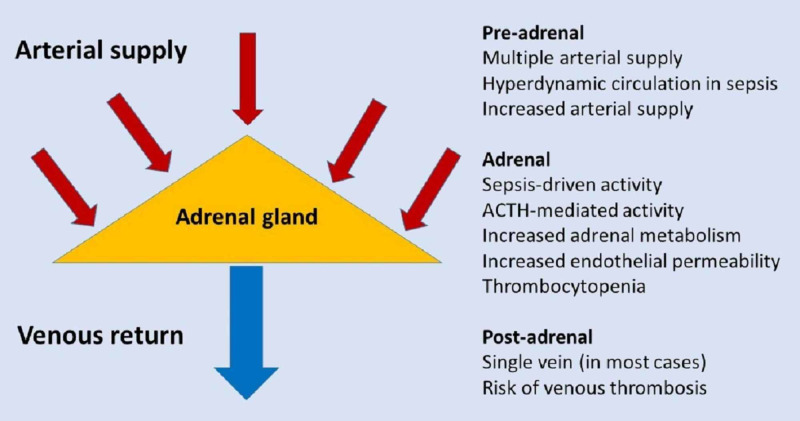
Schematic diagram showing the possible mechanisms involved in spontaneous adrenal hemorrhage ACTH, adrenocorticotropic hormone

The arterial supply to each adrenal gland arises from the aorta, the renal artery, and the inferior phrenic artery, whereas venous return often occurs via only one central vein into the inferior vena cava on the right and via the left renal vein on the left. It is possible that the anatomical architecture of the adrenal gland circulation could contribute to the occurrence of hemorrhage in the context of severe hyperdynamic circulation and increased arterial pressure [10]. See Figure 4 for a schematic representation of the possible mechanisms responsible for adrenal hemorrhage.

Systemic lupus erythematosus (antiphospholipid antibody syndrome, SLE) remains a risk factor for the development of adrenal hemorrhage as was with this case and several reported cases in the literature. The pathogenesis is unclear. However, it has been proposed that the rich cholesterol-containing adrenal glands attract antiphospholipid antibodies, which cause both hemorrhagic and thrombotic destruction [2].

Anticoagulant therapy and heparin-induced thrombocytopenia are risk factors for the development of bilateral adrenal hemorrhage [11]. Therefore, the importance of close monitoring of anticoagulant therapy during sepsis cannot be overemphasized. Also, the simultaneous use of specific antibiotic agents can affect the metabolism of anticoagulants (e.g., warfarin), emphasizing the need for strict monitoring.

Clinical features of adrenal hemorrhage are those of acute adrenal insufficiency, which may include acute abdominal pain, fever, vomiting, weakness, hypotension, and coma. It usually occurs in the context of severe sepsis, particularly when conventional treatment fails to produce any response. Laboratory investigations may reveal a picture compatible with adrenal failure, such as hyponatremia, hyperkalemia, leukocytosis, eosinophilia, and anemia [12]. Heightened awareness and clinical suspicion are required to diagnose this life-threatening condition. Risk factors that should alert clinicians to the diagnosis of adrenal hemorrhage include the lack of response to conventional sepsis treatment, unexplained abdominal pain, and the presence of coagulopathy. It has been suggested that the presence of eosinophilia in the context of severe sepsis can be an indication of adrenal insufficiency [13]. CT scanning is sufficient to confirm the radiological diagnosis in most cases of adrenal hemorrhage [3].

The patient, in this case, had several features in keeping with adrenal insufficiency secondary to adrenal hemorrhage, namely history of abdominal pain, nausea, intractable hypotension, anemia, coagulopathy, thrombocytopenia, mild hyponatremia and inappropriately low serum cortisol levels in the presence of sepsis. She did not have eosinophilia or hyperkalemia. The early morning serum cortisol level was extremely low and therefore, a short Synacthen® test was not carried out prior to commencing emergency corticosteroid therapy. The patient did not have any other cause for adrenal insufficiency such as Addison's disease or prolonged corticosteroid usage. The CT scan findings were not characteristic of adrenal gland tuberculosis or malignancy. It is difficult to ascertain when the hemorrhage occurred but we suspect it was sometime pre-admission as evidenced by the drop in hemoglobin level. The adrenal failure would have ensued shortly afterwards and would have been exacerbated by the sepsis related to the ongoing pneumonia and urinary tract infection.

In a severely ill patient suspected of having adrenal insufficiency, a stimulated increment in the cortisol level below 248.3 nmol/L (after administration of 250 µg of cosyntropin® or synacthen®) or a random total cortisol level below 275.9 nmol/L is diagnostic [4]. Our patient was severely ill and had an inappropriately low random total cortisol level at that time. One would have preferred to do a short Synacthen® test to confirm a diagnosis of adrenal insufficiency but this is not always practical during the acute illness stage when faced with a radiological diagnosis of severe bilateral adrenal hemorrhage. It is safer to commence intravenous steroid therapy, especially when a blood sample for random serum cortisol has already been obtained for laboratory analysis. 

Recovery of adrenal gland function after adrenal hemorrhage is poorly documented in the literature but has been reported [14-16]. A repeat CT scan revealed resolution of the hematomas in our patient, but adrenal function did not recover. Further research is required to assess the recovery of adrenal function after bilateral adrenal hemorrhage. This would aid in providing practice guidelines for the long-term follow-up of affected patients, especially concerning follow-up imaging and regular assessment of adrenal function to identify those patients that may be able to stop long term corticosteroid replacement therapy.

## Conclusions

This is a report of a 76-year-old female who developed adrenal insufficiency secondary to bilateral adrenal hemorrhage in the setting of sepsis and other risk factors. This case emphasizes the importance of heightened awareness and clinical suspicion of this life-threatening condition when faced with a similar scenario.
